# Caregiver stress in stroke survivor: data from a tertiary care hospital -a cross sectional survey

**DOI:** 10.1186/s40359-014-0049-9

**Published:** 2014-11-20

**Authors:** Qurat Ul Ain, Nayab Zaheer Dar, Arsalan Ahmad, Saad Munzar, Abdul Wahab Yousafzai

**Affiliations:** Shifa College of Medicine, Shifa Tameer-e-Millat University, Pitras Bukhari Road, H-8/4, Islamabad, Pakistan; Associate Professor, Division of Neurology, Shifa College of Medicine and Shifa International Hospital, Shifa Tameer-e-Millat University, Pitras Bukhari Road, H-8/4, Islamabad, Pakistan; Division of Neurology, Shifa International Hospital, Shifa Tameer-e-Millat University, Pitras Bukhari Road, H-8/4, Islamabad, Pakistan; Assistant Professor, Department of Psychiatry, Associate Professor, Shifa College of Medicine and Shifa International Hospital, Shifa Tameer-e-Millat University, Pitras Bukhari Road, H-8/4, Islamabad, Pakistan

**Keywords:** Caregiver stress, Stroke, Pakistan

## Abstract

**Background:**

A principal caregiver (CG) is directly affected by patient’s health problems that lead to CG strain. Pakistan has an estimated 4.8% of the population suffering from strokes. The study objective was to evaluate the caregiver level of stress and the factors which make CGs more prone to stress and also to identify the predictive role of factors such as age, sex, educational, marital status on their burden.

**Methods:**

This was a cross-sectional survey. 112 Participants were chosen on the basis of being directly involved in the care of patient and able to give consent for the study. Stroke patients had to have a more than 1 month history of stroke and treated in a tertiary care hospital. The severity of stress was rated using the Modified Caregiver Strain Index (MCSI).

**Results:**

Out of a total of 112 stroke patients and their caregivers, 12 were exempted. Most of the CGs were between the ages 30–39 (48%) and male (70%). Out of the males, most were sons (89%). None of the female CGs was employed. The mean MCSI score was 13.8. Gender, age, marital status, and duration of care all did not have a significant effect on the total (P = 0.640, 0.848, 0.839, 0.110 respectively). Female gender (P = 0.0075) was a factor leading to increased emotional adjustments. Single CGs had increased changes in personal plans (P = 0.014), and married CGs found the behaviour of the patients less upsetting (P = 0.0425). There was no significant difference between the total (P = 0.906) or individual components between daughters and daughter-in-laws. Increased duration of care was significantly associated with decrease level of sleep disturbance (P = 0.026), physical strain (P = 0.050) and other demands on time (P = 0.044). Increase age of CG was associated with an increase feeling of being overwhelmed (P = 0.027).

**Conclusion:**

There is a need to identify the factors responsible for major CG stress by conducting similar studies and to define structured intervention for evaluating and preventing problems of caregivers.

## Background

Neurological damage accounts for approximately 40% of cases of severe disability (in which individuals require daily help) and for the majority of cases of complex disability resulting from combinations of physical, cognitive and behavioral impairments (Greenwood 2001).

The overall burden of neurological diseases in lower income countries like Pakistan ranges from 4-5% compared to 11% in higher income countries (Neurology on the Global Health Agenda 2007). The disability related to neurological diseases is higher than Ischemic heart diseases, malignancies or HIV. Stroke being the 3rd most common cause of death is related to more than half of the death and disability in these diseases.

Because of the changing lifestyles and urbanization of the population, including an increase in risk factors like smoking, obesity, physical inactivity, high blood pressure and cholesterol, the burden of stroke rather than decreasing like in the Western countries has increased in the South Asian countries.

According to World Health Organization estimates, 5.5 million people died of stroke in 2002, and roughly 20% of these deaths occurred in South Asia. Contrary to decline in the incidence of the disease in the Western population, the burden of the disease in South Asian countries (India, Pakistan (Khealani et al. 2008), Bangladesh, and Sri Lanka) has inclined and is expected to rise.

Pakistan being the 6th most populous country with an estimated population of 187 million has an estimated 4.8% of the population suffering from Stroke translating to 8,976,000 individuals (Jafar 2006). A recent community survey in Kolkata, carried out by the Indian Council of Medical Research, showed the average annual incidence of stroke as 145 per 100,000 persons per year (Das et al. 2007). These rates are also much higher than those reported previously from other parts of India. In China, the total average age adjusted incidence of first-ever stroke ranged from 116 to 219 per 100 000 per year (Liu et al. 2007).

Brain-injuries, such as stroke, have been shown to significantly affect the psychological well-being of patient’s family members (Godwin et al. 2013). This disease disables individuals and places considerable burden not only on the family of the individual but on the community as a whole. Caring for stroke patients often puts considerable strain on the caregivers. It is therefore important to understand the factors affecting the caregiver’s strain especially since they are prone to psychological and physical illnesses along with a major financial burden. The prevalence of depression is reported to be higher in caregivers than in stroke survivors, with an estimated range from 30–52% of depression in stroke caregivers (Gonzalez & Bakas 2013).

Caregivers can be considered the second victims of the disease, they often take on this role under sudden and extreme circumstances, with minimal preparation and little guidance and support from healthcare systems (Bartolo et al. 2010). A qualitative study of caregivers of stroke survivors found caregiver distress to begin soon after the initiation of caregiving and to last for more than a year after the stroke. In addition, they found caregivers to report psychological distress 2.5 times more than non-caregivers (Godwin et al. 2013).

Recent research suggests that much of the increased risk for poor caregiver outcomes is due to the amount of mental or emotional strain associated with providing care. Caregivers who subjectively reported a high amount of strain also reported poorer physical functioning, fewer social contacts, and more emotional distress than other caregivers (Clay et al. 2013).

A caregiver has responsibilities to not only look after the disabled individual but also to make adjustments to his/her life. Need of a stroke survivor may vary from being physical (help walking, carrying from bed to toilet), in communication (verbal and nonverbal cues to other family members), nursing (feeding, personal hygiene), emotional support (handling disruptive behaviour) along with an overwhelming financial responsibility.

So the objective of this study were to evaluate the level of caregiver stress of stroke patients and the factors which made them more prone to stress and also to identify the predictive role of factors such as age, sex, education, marital status on burden.

## Methods

This was a cross sectional survey of caregivers of stroke patients. After approval from our Institutional Review Board (IRB) of Shifa International Hospital/Shifa College of Medicine, data was collected from 15th November 2012 to 15th February 2013.

The severity of stress was rated using the Modified Caregiver Strain Index (MCSI). It is a 13 question tool that measures stress related to care provision. MCSI assesses the 5 elements in care giver strain. These include physical, psychological, social, personal and financial (Lisa 2013). Scoring is two points for ‘Yes’ and one for ‘Sometimes’ and zero for ‘No’. The higher the score, the higher the level of caregiver stress. The tool is brief, easy to administer and can even be self completed by the caregiver. Its results can be used to pinpoint the causes and degree of strain, as well as changes in strain over time, so interventions can be implemented before caregiver’s health suffers significantly (Thornton & Travis 2003).

### Operational definitions

1. Stroke is defined as rapidly developing signs of focal disturbance of cerebral function lasting more than 24 hours with no apparent non vascular cause (Thorvaldsen et al. 1997).
2. A principal caregiver was defined as “any person who, without being a professional or belonging to a social support network, usually lives with the patient and, in some way, is directly implicated in the patient’s care or is directly affected by the patient’s health problem” (Godwin et al. 2013).
3. Moderate disability was defined as the patient not able to move without support.
4. Severe disability was defined as any patient being bedridden, not being able to feed himself.

### Inclusion criteria

The patients suffering from either haemorrhagic or ischemic stroke with more than one month history of the disease with moderate to severe disability. All of the above stated patients were treated in a tertiary care hospital; Shifa International Hospital Islamabad. Caregivers (CGs) were included if they were directly involved in the care of the patient; able to give consent for the study.

### Exclusion criteria

If the CGs were not directly related to the patient, if more than one caregiver was involved or if they were being paid for their services.

Patient disease related information was collected by using their medical documents available, that included; date of birth, sex, type of stroke and duration of illness. After giving a brief summary to the CGs on the purpose of the study consent was obtained from CGs^.^Data was collected both by telephonic interview and also by face to face interviewing done when the CGs came to the hospital for the patient’s follow-up or were admitted for other causes like infections, cardiovascular or endocrine reasons.

### Statistical analysis

Analyses were conducted to evaluate the effect of gender, marital status and age of CGs as well as their duration of care on total and individual components of the MCSI score. Results from all analyses were evaluated at α = 0.05.

For gender and marital status, we performed individual Wilcoxon-Mann–Whitney analyses to determine if significant differences in total MCSI score existed between male and female CGs, or single and married CGs.

(Total MCSI score in male CGs vs Total MCSI score in female CGs, Total MCSI score in single CGs vs Total MCSI score in married CGs) Similarly, we performed individual Wilcoxon-Mann–Whitney analyses to determine if significant differences in each of the individual component scores of the MCSI existed between male and female CGs, or single and married CGs.

We assessed correlations between age of CGs and total MCSI scores, as well as between the duration of care by CGs and total MCSI scores using the Spearman correlation, given that the scales were ordinal. Similarly we assessed the correlations between age of CGs and each of the individual component scores of MCSI, as well as between the duration of care by CGs and each of the individual component scores of MCSI using the Spearman correlation. The correlation coefficients (r) were interpreted as r >0.3 weak correlation; 0.3 < r >0.7 moderate correlation; r >0.7 strong correlation.

The data was analysed on SPSS version 20 for factors such as age, sex, education, employment status and marital status. Due to the lack of prior local studies in this subject, no specific statistical assumptions were made and the sample-size was arbitrarily determined.

## Results

**Figure 1:**
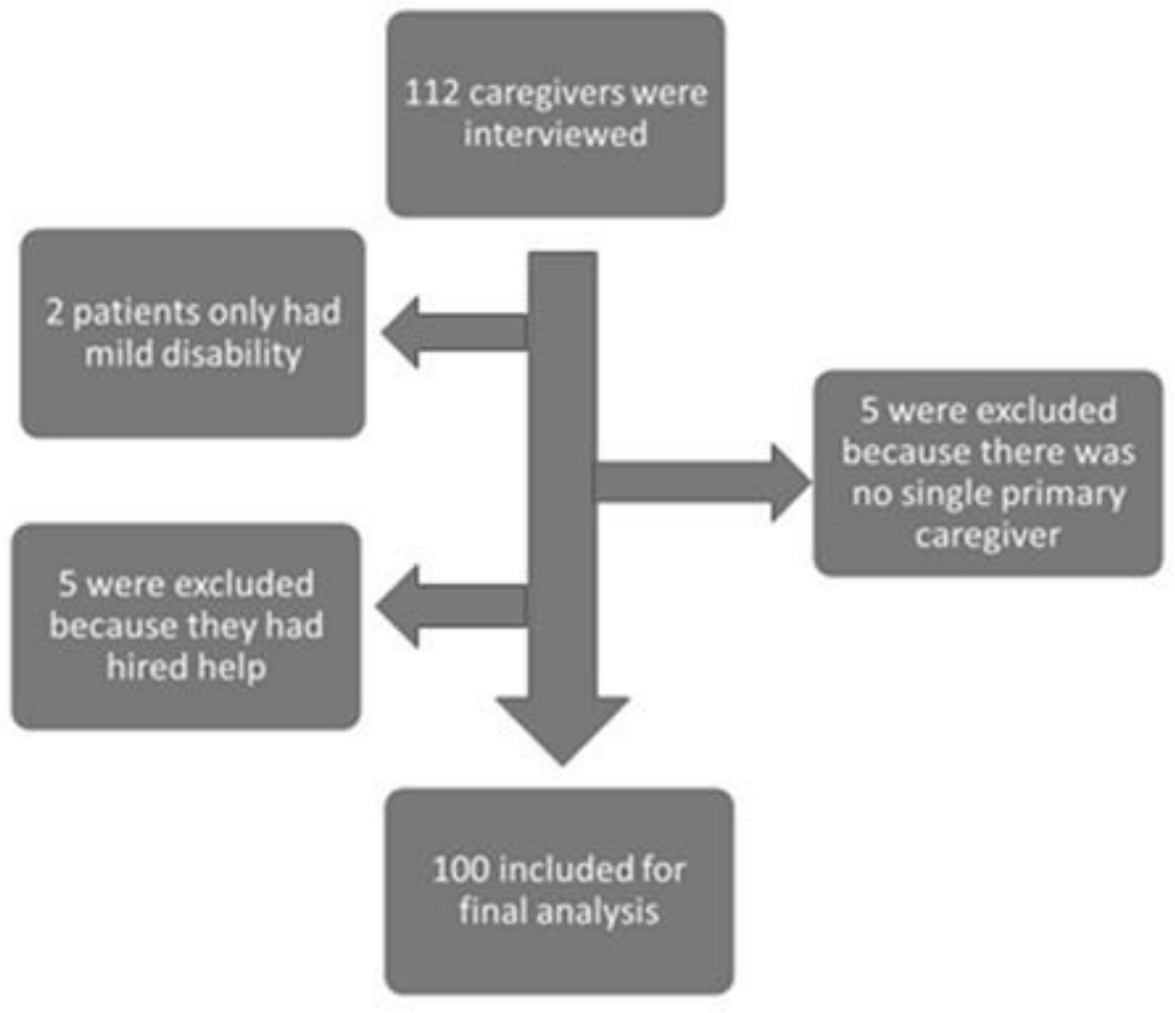
**Exclusion of participants.**

During the period of the study 112 caregivers were interviewed; from which 5 were excluded because they were hired help, 2 patients only mild disability and were not dependent on any help from the CGs, 5 more were excluded because there was no primary caregiver and more than one person was taking care of the patient in different shifts. Thus a total of 100 CGs were included for the final analysis (Figure 1).

70% of the caregivers were male, out of which 89% were sons and other 11% included husbands, brothers, nephews, grandsons. Out of the 30% of female caregivers, 57% were daughters 37% were daughter in laws and other 6% included relationships like nieces, granddaughters.

**Figure 2:**
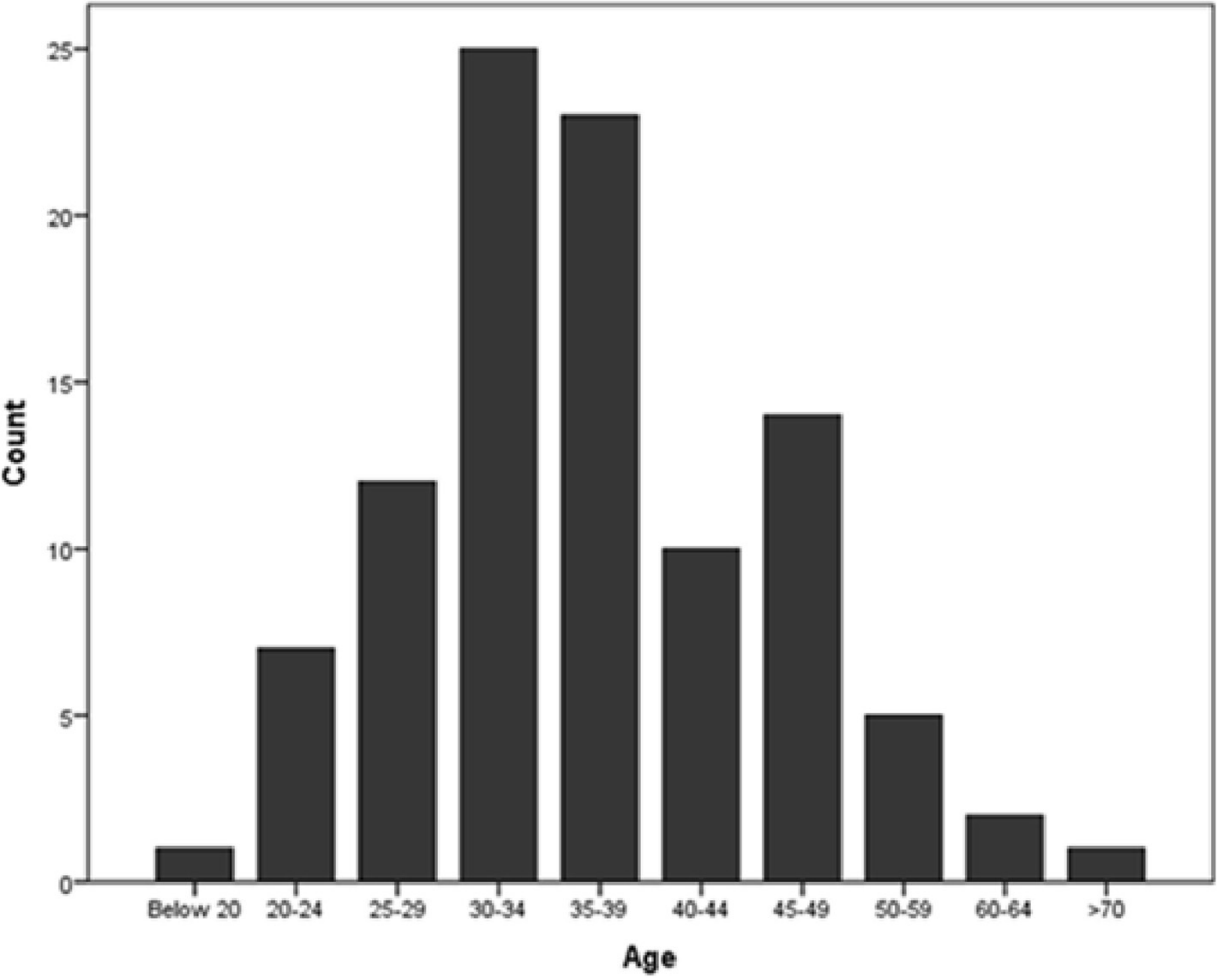
**Bar diagram representing ages of caregivers.**

The mean age of CGs was 30–39 which accounted for 48% of the total (Figure 2).

On analysing the employment status, 76% of the males were employed, most of them as shopkeepers, teachers or self-employed. They usually had to quit their jobs or skip days from work due to sleep deprivation; taking the patients for follow up appointments and taking care of the patients at home. This affected the caregivers financially also none of the females were employed mainly due to the norms in Pakistan and only 13% were students.

While calculating mean total score we found that gender, age, marital status, and duration of care all did not have a significant effect on the total (P = 0.640, 0.848, 0.839, 0.110 respectively) with not statistically significant p values. Female gender (P = 0.0075) was a factor leading to increased emotional adjustments.

Single CGs had increased changes in personal plans (P = 0.014), and married CGs found the behaviour of the patients less upsetting (P = 0.0425) (Table 1).

**Table 1:** **Comparison of male vs. female, single vs. married stress responses to factors**

	**Wilcoxon-Mann–Whitney** ***U*** **-test (single-sided)**	
	**(Male/Female)**	**(Single/Married)**
1. My sleep is disturbed	0.315	0.290
2. Caregiving is inconvenient	0.149	0.059
3. Caregiving is a physical strain	0.219	0.202
4. Caregiving is confining	0.202	0.344
5. There have been family adjustments	0.837	0.262
6. There have been changes in personal plans	0.093	0.014
7. There have been other demands on my time	0.158	0.376
8. There have been emotional adjustments	0.007	0.097
9. Some behavior is upsetting	0.257	0.043
10. It is upsetting to find the personal I care for has changed so much from his/her former self	0.188	0.132
11. There have been work adjustments	0.000	0.267
12. Caregiving is a financial strain	0.291	0.194
13. I feel completely overwhelmed	0.114	0.133

The mean MCSI score was 13.8. Single CGs had more mean total score of 14.05 than married couples with 13.78. CGs of 30–39 years had more stress score of 15.80 compared to other ages (Table 2).

**Table 2:** **Relation of mean total score with factors affecting CGs**

		**Mean total score**	**p value**
Gender	Male	13.94 (±5.793)	0.640^a^
	Female	13.57 (±5.056)	
Marital			0.839^a^
Status	Single	14.05 (±5.689)	
	Married	13.78 (±5.562)	
Age	<20	11.00 (±0.000)	0.848^b^
		12.57 (±4.276)	
	20-24	11.25 (±4.751)	
		15.80 (6.076)	
	25-29	15.23 (±5.667)	
	30-34	11.70 (±4.218)	
		13.07 (±6.330)	
	35-39	13.80 (±4.604)	
		10.50 (±4.905)	
	40-44	13.00 (±0.000)	
	45-49	17.33 (±3.502)	0.055^b^
	50-59	14.17 (±5.015)	
		13.27 (±5.942)	
	60-64		
	>70		
	1-3 months		
	3-6 months		
	>6 months		

^a^ = Wilcoxon-Mann–Whitney *U* Test (95% confidence).

^b^ = Spearman’s Correlation (95% confidence).

There was no significant difference between the total (P = 0.906) or individual components between daughters and daughter-in-laws.

When the Spearman’s correlation was applied it was found that increased duration of care was significantly associated with decrease level of sleep disturbance (P = 0.026), physical strain (P = 0.050) and other demands on time (P = 0.044) (Table 3). The consistent decrease in caregiver burden over time found in our study may be related to adaptation to the caregiving role. In a qualitative study which followed family caregivers over an 18-month period, the caregivers reported that caregiving became more routine and that their expertise and competence grew over time (Brereton & Nolan 2000).

**Table 3:** **Comparison of duration of care to factors affecting CGs; age of Caregiver relationship to factors affecting CGs**

	**Spearman’s correlation**			
	**Duration of care**		**Age of caregiver**	
	**Correlation coefficient**	**p value (1-tailed)**	**Correlation coefficient**	**p value (1 = tailed)**
1. My sleep is disturbed	−0.194	0.026	−0.057	0.285
2. Caregiving is inconvenient	−0.132	0.095	+0.141	0.081
3. Caregiving is a physical strain	−0.165	0.050	+0.012	0.453
4. Caregiving is confining	−0.022	0.415	−0.009	0.464
5. There have been family adjustments	−0.085	0.201	−0.064	0.265
6. There have been changed in personal plans	+0.001	0.495	−0.130	0.099
7. There have been other demands on my time	−0.171	0.044	−0.067	0.255
8. There have been emotional adjustments	−0.010	0.459	+0.055	0.293
9. Some behavior is upsetting	−0.096	0.172	−0.102	0.155
10. It is upsetting to find the person I care for has changed so much from his/her former self	−0.052	0.302	−0.095	0.174
11. There have been work adjustments	−0.127	0.105	−0.102	0.157
12. Caregiving is a financial strain	−0.077	0.222	+0.129	0.101
13. I feel completely overwhelmed	−0.083	0.206	+0.0193	0.027

Depressive symptoms are common among patients and spouse in the early stages after stroke (Berg et al. 2005). In these stages, the patients’ future disability is uncertain, and uncertainty and anxiety are powerful stressors (McCullagh et al. 2005; Schlote et al. 2006). Increase age of CG was associated with an increase feeling of being overwhelmed with a positive correlation of +0.193 (P = 0.027).

## Discussion

Stroke is a major non-communicable disease. It is the most common cause of mortality and a significant cause of adult disability. Stroke may also compromise cognitive, mood, functional abilities and quality-of-life. It also results in caregiver burden and economic stress at individual, familial and national level (Sujata & Shyamal Kumar 2013). Stroke creates a burden on the whole family due to joint family system in Pakistan, where parents, spouse and children and other in laws live together under one roof.

Financial difficulties are compounded by limited employment opportunities for stroke survivors who are aged or sole earners in the family, the possibility of job retrenchment because of disability or long absenteeism or both, and continuing expenses for medicine and physiotherapy. The financial worries were more common among slum dwellers and less educated CG, possibly because of limited financial capability (Das et al. 2010).

Factors associated with caregiver burden that can lead to caregiver stress include “the relationship quality between caregiver and patient, the patient’s cognitive ability, behavioural and psychological symptoms displayed by the patient, caregiver gender, and adverse life events” (Campbell et al. 2008).

Stroke causes major illness burden on the family through different means, financial social, psychological ways. As soon as the patient develop stroke they are taken to the nearby clinic or hospital either government or private. So the medical care facility charges, frequent follow-ups and transportation charges all leads to continuous source of mental and financial stress for the families due to low socioeconomic state of people in Pakistan.

We choose to use MCSI index as it assesses the 5 elements in care giver strain. These include physical, psychological, social, personal and financial. Scoring is two points for ‘Yes’ and one for ‘Sometimes’ and zero for ‘No’. The higher the score, the higher the level of caregiver stress.

In our study, 70% of the caregiver comprises of male members, who were experiencing the stress as sole figures in the family. Due to family setup in Pakistan 89% sons were caregivers as daughter if married were living with their husband’s family. Out of 30% female, 57%were unmarried daughters.

Family caregivers may be motivated to provide care for several reasons: a sense of love or reciprocity, spiritual fulfilment, a sense of duty, guilt, social pressures, or in rare instances, greed (Eisdorfer 1991). Caregivers who are motivated by a sense of duty, guilt, or social and cultural norms are more likely to resent their role and suffer greater psychological distress than caregivers with more positive motivations (Pyke & Bengston 1996).

Caregivers face many obstacles as they balance caregiving with other demands, including child rearing, career, and relationships. They are at increased risk for burden, stress, depression, and a variety of other health complications. The effects on caregivers are diverse and complex, and there are many other factors that may exacerbate or ameliorate how caregivers react and feel as a result of their role (Brodaty & Donkin 2009).

The effects on caregivers are diverse and complex, and there are many other factors that may exacerbate or ameliorate how caregivers react and feel as a result of their role (Lo Giudice et al. 1999).

Caregivers often lack social contact and support and experience feelings of social isolation (Lo Giudice et al. 1999).

Caregivers tend to sacrifice their leisure pursuits and hobbies, to restrict time with friends and family, and to give up or reduce employment (Leong et al. 2001; Brodaty & Hadzi-Pavlovic 1990). Caregivers who are more satisfied with their social interactions show fewer negative psychological symptoms (Lowery et al. 2000). Interventions may assist. One psychosocial intervention significantly increased the number of support persons for caregivers, their satisfaction with their support network, and the assistance they received with caregiving, compared with controls (Serrano-Aguilar et al. 2006).

Our study has some limitations: Due to the lack of prior local studies in this subject, no specific statistical assumptions were made and the sample-size was arbitrarily determined; the small sample size needs to be kept in mind while interpreting the findings. Secondly, the care givers were selected randomly through the record available in hospital, so it cannot be applied to the whole population of Pakistan, further study is required.

In our society and culture, joint family system helps in dividing the burden and supporting the patients together to improve the environment and managing the condition.

Indeed there is evidence that intervention targeting caregivers can decrease their level of stress, depression and anxiety (Coon et al. 2003; Gerdner et al. 2002; Gitlin et al. 2003) and increase their sense of control and their ability to cope with the burdensome experience of care giving: (Lowery et al. 2000) the potential benefits of person-cantered intervention require further evaluation (Coon & Evans 2009).

## Conclusion

Most stress faced by the caregivers is due to untrained care giving, sleep disturbances and disturbances in managing their own family life.

High quality stroke services are not widely available and there is an urgent need of improvement in infrastructure to conduct well-designed epidemiological studies, create awareness in general public regarding stroke and improve capacity building in order to meet the future challenges (Khealani et al. 2008).

Caregivers and family members should be trained and educated about home care before the patient is discharged. They should be counselled about management of their own stress and personal life and health in a better way. This will help in reducing their anxiety and stress levels, and bring better outcomes for both, the patient and the caregiver.
